# Supplementary material for: Tamoxifen mechanically reprograms the tumor microenvironment via HIF‐1A and reduces cancer cell survival
Source: EMBO Rep. 2018 Dec 12;20(1):e46557. doi: 10.15252/embr.201846557 (PMC6322388; doi:10.15252/embr.201846557)
Supplement: Supplementary file 1 — Appendix [file EMBR-20-e46557-s001.pdf]

Tamoxifen mechanically reprograms the tumor microenvironment and the survival of cancer cells via HIF-1A

Ernesto Cortes, Dariusz Lachowski, Benjamin Robinson, Muge Sarper, Jaakko S. Teppo, Stephen D. Thorpe, Tyler J. Lieberthal, Kazunari Iwamoto, David A. Lee, Mariko Okada-Hatakeyama, Markku T. Varjosalo, and Armando E. del Río Hernández

Appendix – Table of content

- **Appendix Figure S1**. Changes in PSCs expression for the HIF and VEGF family of genes……………………………………………………………………………………**Page 2**
- **Appendix Figure S2.** Map of HIF-1A related differentially expressed genes in PSCs……………………………………………………………………………………**Page 3**
- **Appendix Figure S3.** Western blots for HIF-1A and total protein for PSCs control (Con) and treated with tamoxifen (Tam) ……………………………………………………**Page 4**
- **Appendix Figure S4.** Knockdown efficiency of siRNA HIF-1A in PSCs ……..……**Page 5**
- **Appendix Figure S5.** Tamoxifen does not affect TIMP-2 levels in PSCs or MMP-9 levels in PSCs and PDAC tissues ……………………………………………..……………**Page 6**
- **Appendix Figure S6.** Matrix stiffness and HIF-1A modulates the effect of tamoxifen in FN levels on PSCs ………………………………..……………………………………**Page 7**
- **Appendix Figure S7**. Matrix stiffness and myosin activation modulates the effect of tamoxifen in HIF-1A levels on Suit-2 cells ………………………………………..……**Page 8**
- **Appendix Table S1.** RPKM values for the analysis of tubulin-related genes (RNA sequencing of control and tamoxifen (tam) treated PSCs. …………………..……**Page 9**
- References for Appendix Figure 2……….………………………......……….**Pages 10-11**

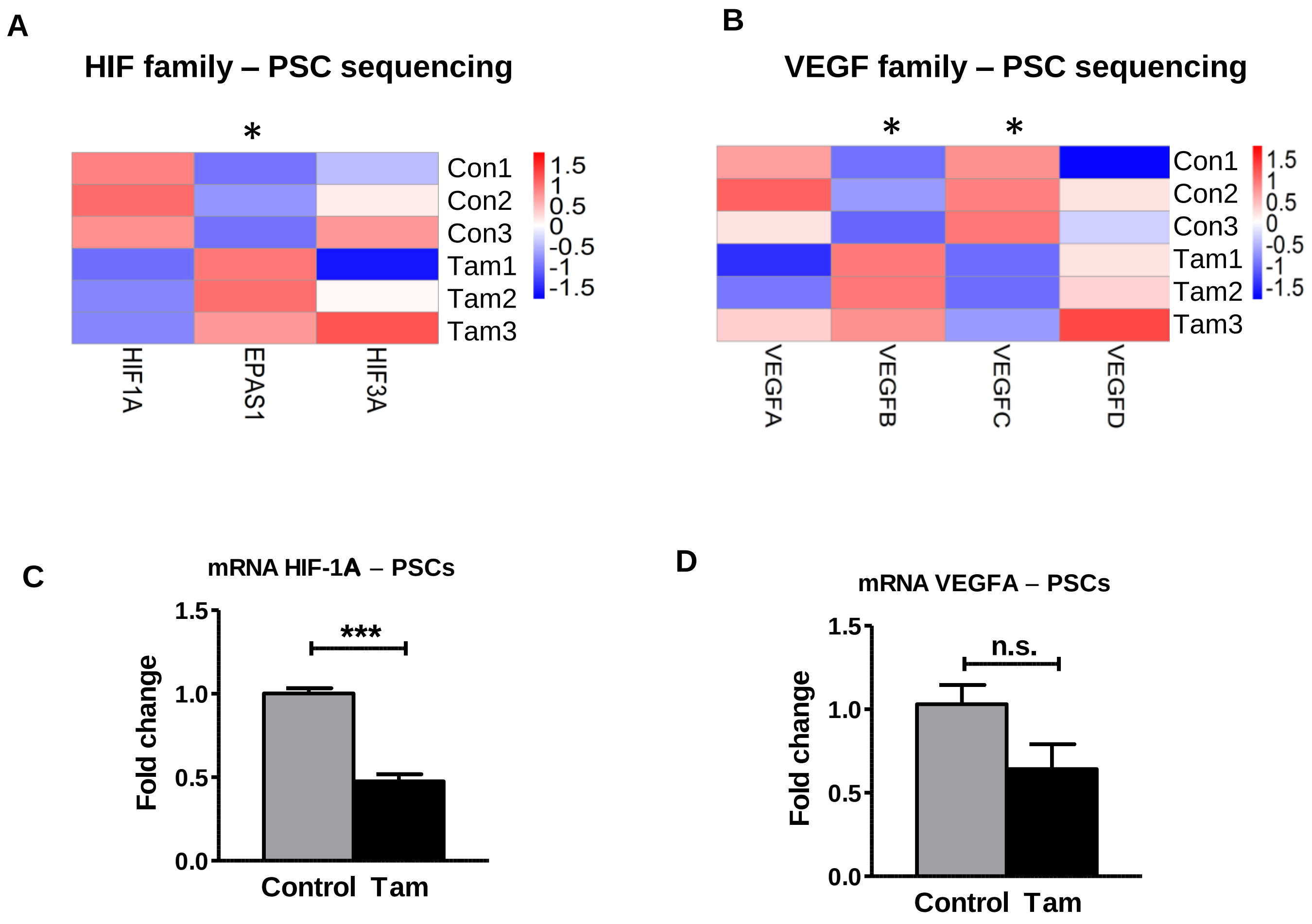

Appendix Figure S1: Changes in PSCs expression for the HIF and VEGF family of genes. (A-B) Expression of HIF and VEGF family genes obtained from RNA-seq data in control and tamoxifen treated PSCs (n = 3 experimental replicates). Expression value was normalized by tubulin family genes. EPAS1 corresponds to HIF-2A. **(C-D)** qPCR levels of HIF-1A and VEGFA in PSCs, normalized to RPLP0 and relative to control. Assay was done for 3 separate experiments. Bars represent mean±SEM. t-test used. * $p<0.05$, *** $p<0.001$

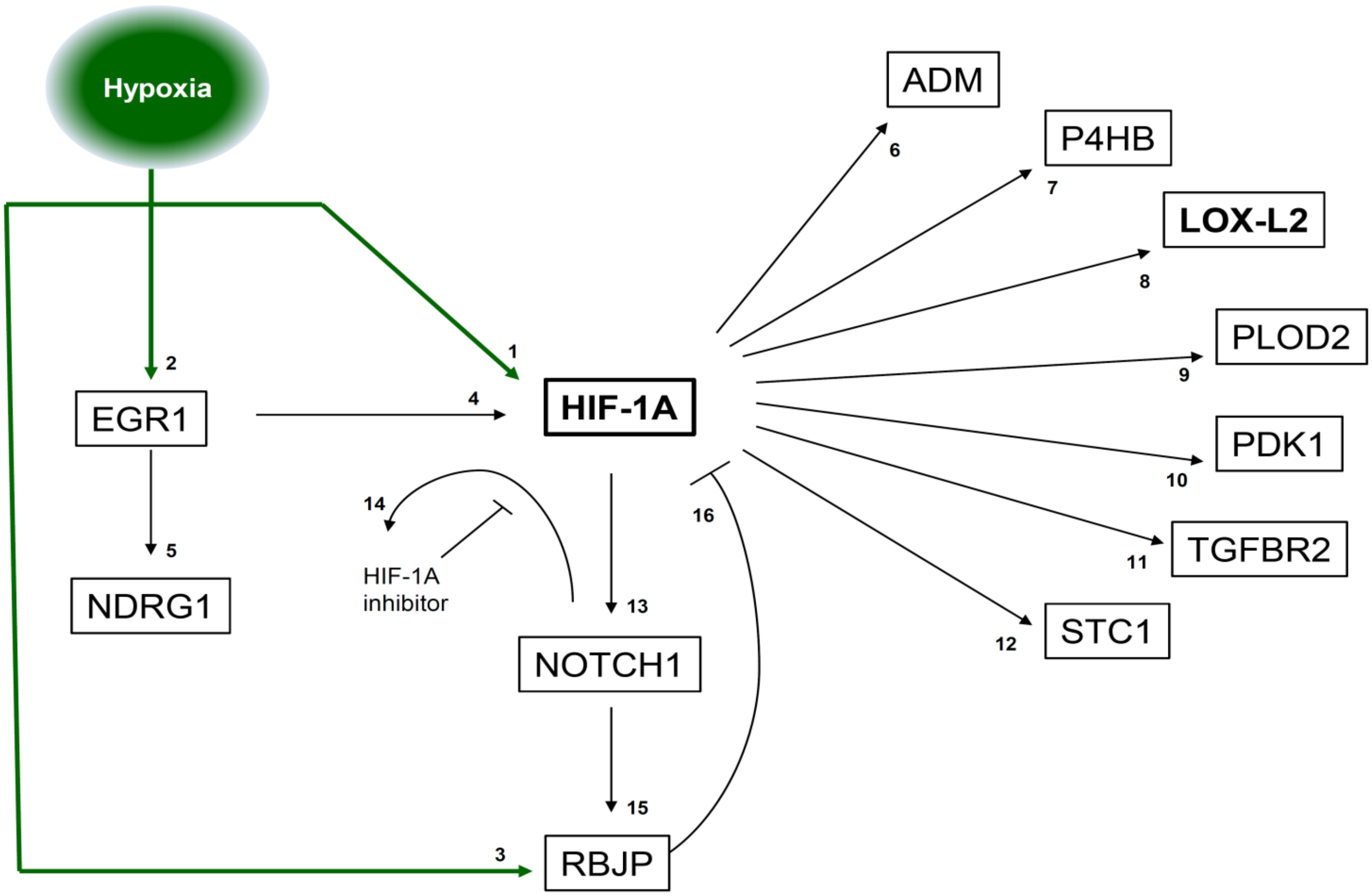

Appendix Figure S2: Map of HIF-1A related differentially expressed genes in PSCs. These genes are taken from the hypoxia downregulated genes in Fig 1E. Hypoxia increases HIF-1A (Ref1), EGR1(Ref2) and RBJP(Ref3). EGR1 positively regulates (Ref4). EGR1 positively regulates NDR1 (Ref5). HIF-1A positively regulates ADM (Ref6), P4HB (Ref7), LOX-L2 (Ref8), PLOD2 (Ref9), PDK1 (Ref10), TGFBR2 (Ref11), STC1 (Ref12), and Notch1 (Ref13). Notch1 may enhance HIF-1A by sequestering HIF-1A away from HIF-1A (Ref14). Notch1 controls RBJP (Ref15). RBJP inhibits HIF-1A (Ref16). References – at the end of the appendix in pages 10-11.

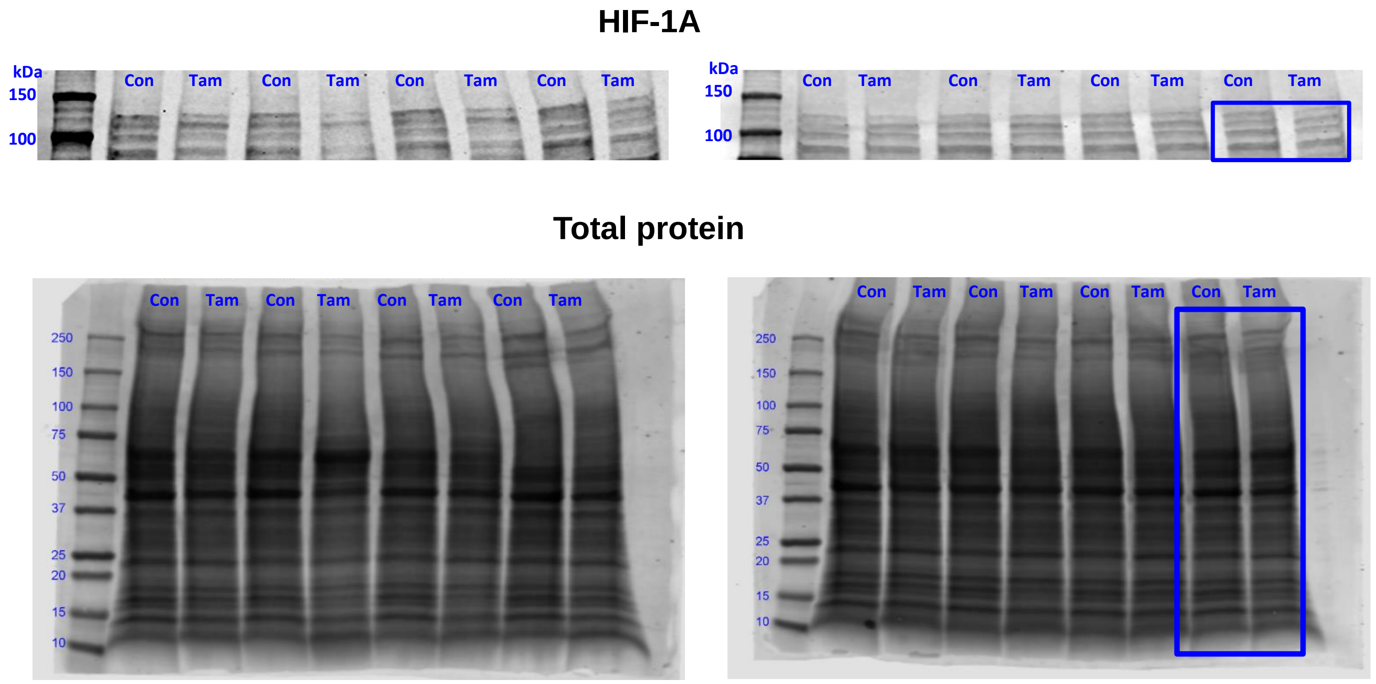

Appendix Figure S3: Western blots for HIF-1A and total protein for PSCs control (Con) and treated with tamoxifen (Tam). Bands presented in main Figure 1J are indicated by blue rectangle.

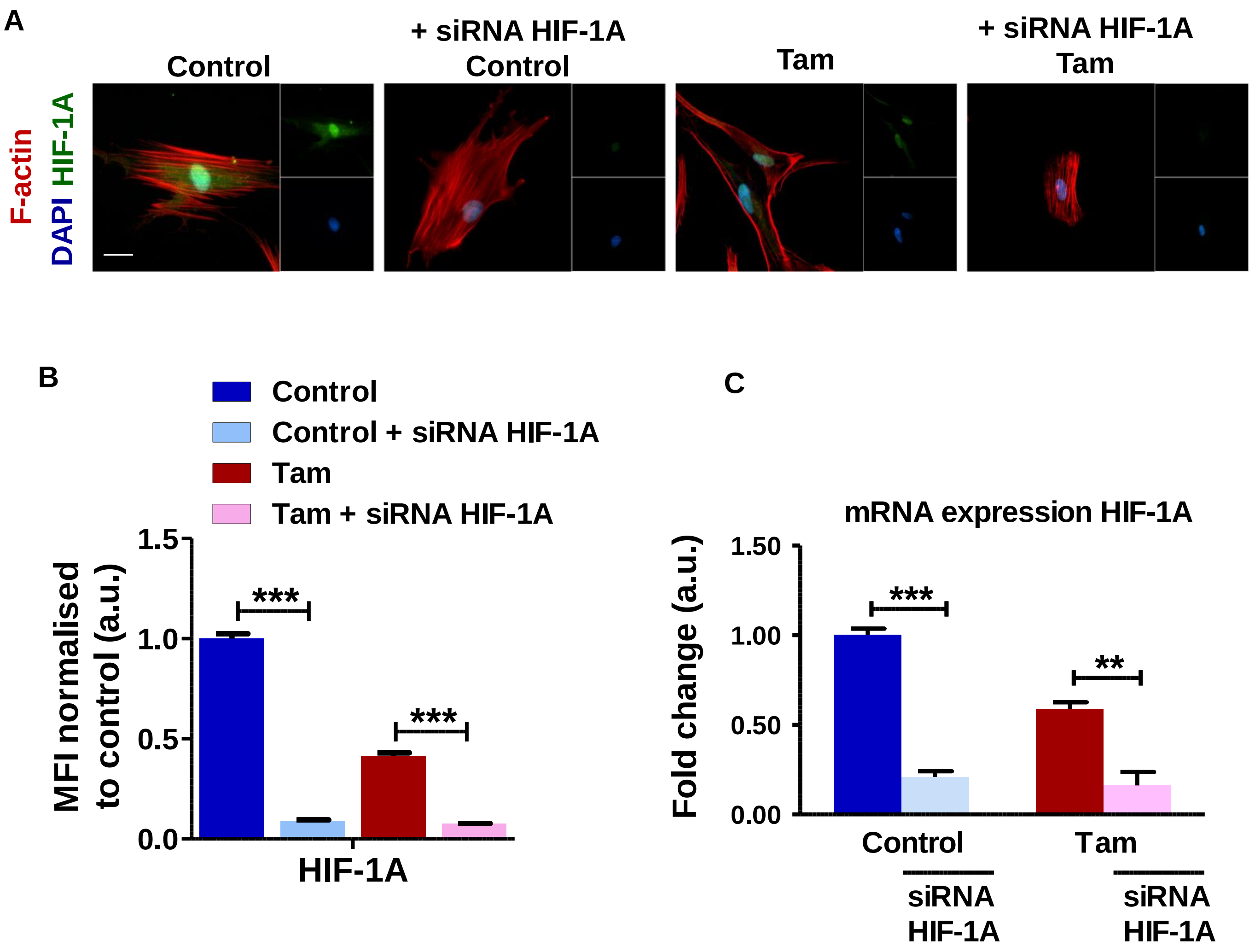

Appendix Figure S4: Knockdown efficiency of siRNA HIF-1A in PSCs. (A) Representative immunofluorescent images of PSCs, scale bar is 20 μm. **(B)** Quantification of fluorescence intensity for images in panel A (n=32 control, 33 control + siRNA HIF-1A, 28 tam, 35 tam + siRNA HIF-1A). **(C)** qPCR levels of HIF-1A in PSCs, normalized to RPLP0 and relative to control. Three biological samples taken in three different experiments. Histogram bars represent mean ± s.e.m.;***P<0.001 (t-test).

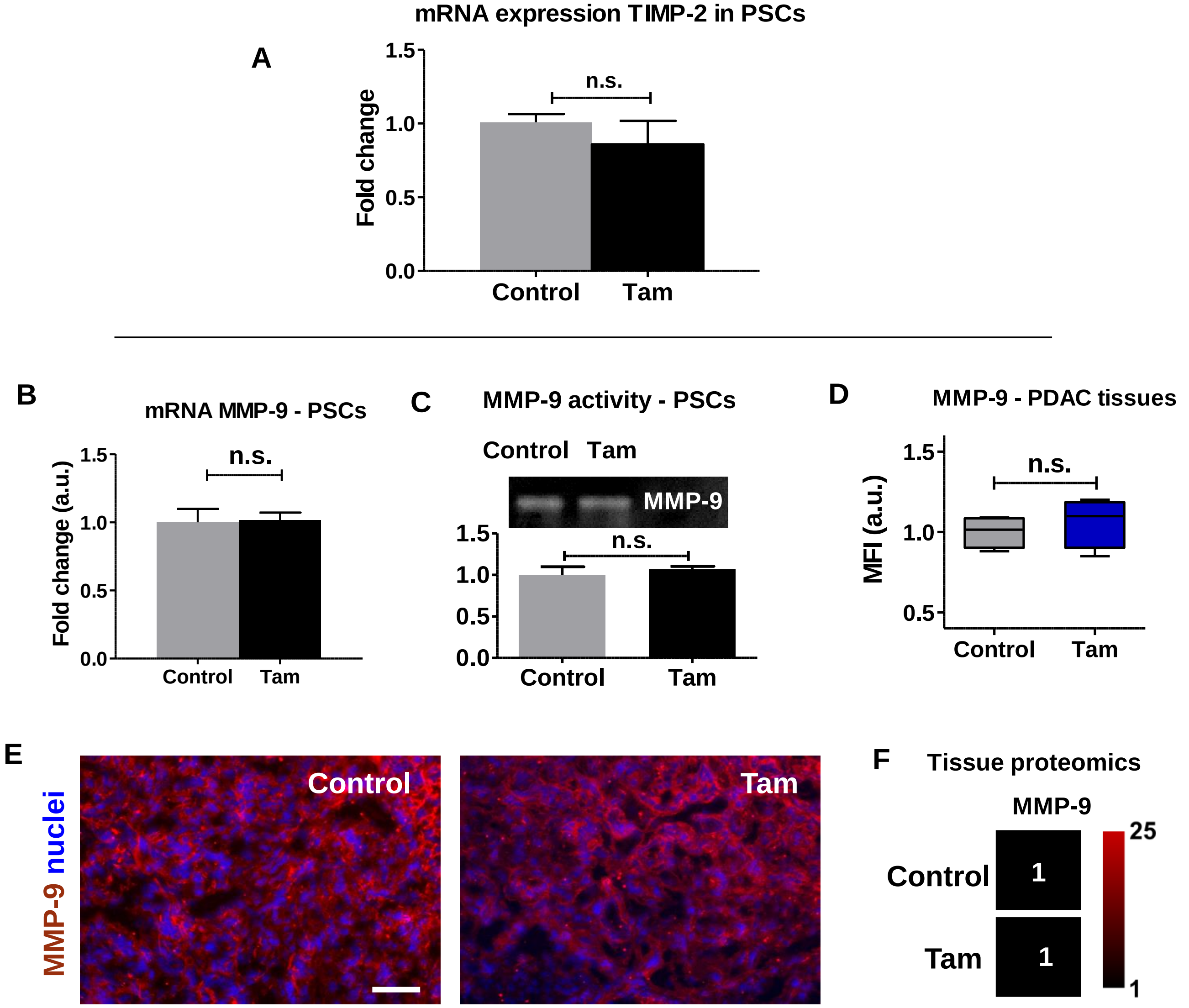

Appendix Figure S5. Tamoxifen does not affect TIMP-2 levels in PSCs or MMP-9 levels in PSCs and PDAC tissues. (A) mRNA levels of TIMP-2 in PSCs, normalized to RPLP0 (60S acidic ribosomal protein P0) **(B)** qPCR levels of MMP-9 in PSCs, normalized to RPLP0 and relative to control. **(C)** MMP-9 activity on control and tamoxifen treated PSCs assayed by gelatin zymography; above signal intensity of the representative bands used for the quantification presented in the plot below. **(D)** Quantification of MMP-9 in PDAC tissues. In the box-and-whisker plot, the central box represents values from the lower to upper quartile. The middle line represents the mean. The vertical line extends from the minimum to the maximum value (n=5 animal per condition, and n≥ 5 sections per animal). **(E)** Immunofluorescence images of PDAC tissues from KPC mice treated with vehicle control, and 5mg of tamoxifen, scale bar 100 μm. **(F)** Relative values of protein levels for MMP-9 in PDAC tumours assessed by proteomic analysis (6 mice per condition and samples were analysed in duplicates). In all cases, bars represent mean±s.e.m.; *P<0.05,**P<0.01,***P<0.001 (t-test).

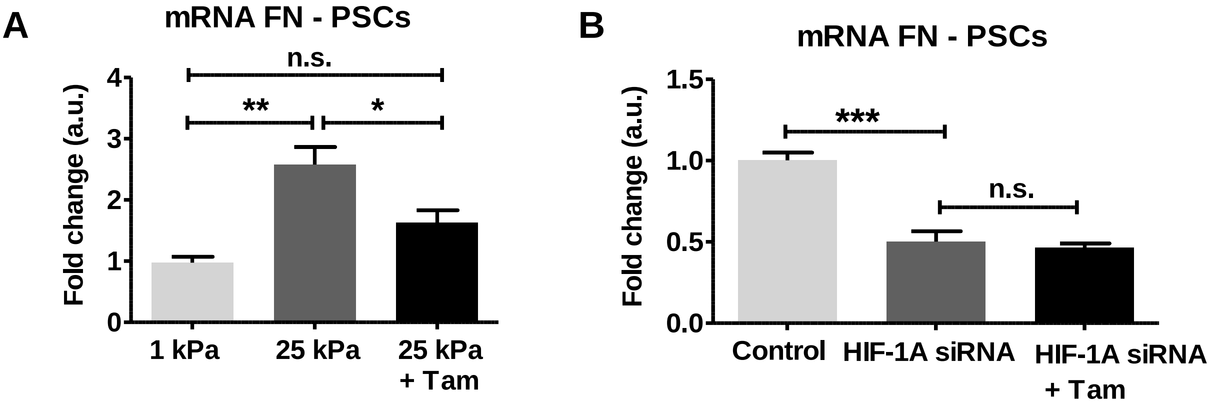

Appendix Figure S6: Matrix stiffness and HIF-1A modulates the effect of tamoxifen in FN levels on PSCs. qPCR levels of FN (fibronectin) in PSCs, normalized to RPLP0 and relative to control. Assay was done for 3 separate experiments. Bars represent mean±SEM. Anova and Tukey's post hoc test used. * $p<0.05$, ** $p<0.01$, *** $p<0.001$, n.s. not significant differences.

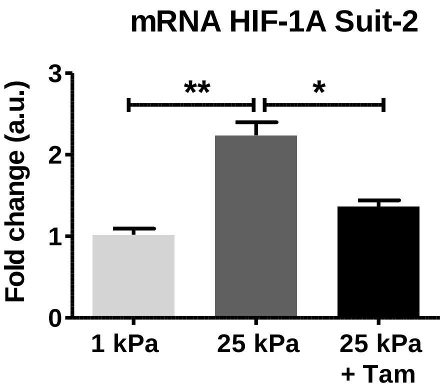

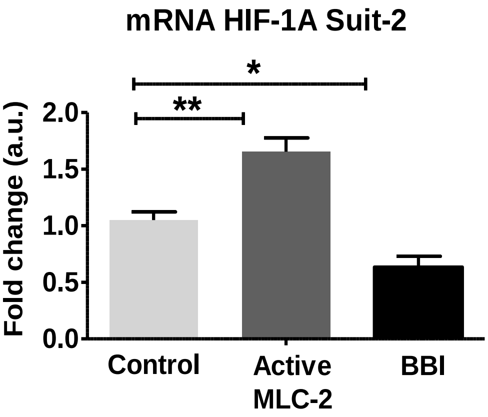

Appendix Figure S7: Matrix stiffness and myosin activation modulates the effect of tamoxifen in HIF-1A levels on Suit-2 cells. qPCR levels of HIF-1A on Suit-2 cells, normalized to RPLP0 and relative to control. BBI is blebbistatin. Assay was done for 3 separate experiments. Bars represent mean±SEM. Anova and Tukey's post hoc test (left) and t-test (right), ***p<0.001, **p<0.01, *p<0.05.

Appendix Table S1: RPKM values for the analysis of tubulin-related genes (RNA sequencing of control and tamoxifen (tam) treated PSCs. p-value is calculated using Mann–Whitney U test. RPKM is reads per kilo-base per million).

Gene	Control1	Control2	Control3	Tam1	Tam2	Tam3	p-value
**TUBA1A**	194.262	183.319	190.522	119.716	124.707	116.274	**0.1**
**TUBA1B**	54.754	53.655	54.773	26.489	30.771	28.752	**0.1**
**TUBA1C**	24.646	23.301	23.715	12.029	12.635	11.770	**0.1**
**TUBA3D**	0.107	0.095	0.111	0.081	0.141	0.056	**0.7**
**TUBA3FP**	0.018	0.045	0.037	0.061	0.097	0.053	**0.1**
**TUBA4A**	6.349	6.031	6.016	2.129	2.436	1.968	**0.1**
**TUBB**	373.137	358.539	371.202	237.472	249.764	230.224	**0.1**
**TUBB1**	0.060	0.043	0.045	0.016	0.016	0.026	**0.1**
**TUBB2A**	23.105	20.528	21.236	13.592	13.787	14.616	**0.1**
**TUBB2B**	0.762	0.673	0.619	0.897	0.658	0.958	**0.4**
**TUBB3**	0.352	0.304	0.320	0.201	0.279	0.136	**0.1**
**TUBB4B**	114.608	115.312	116.636	57.312	57.717	56.737	**0.1**
**TUBB6**	83.327	81.150	81.793	51.310	50.651	50.196	**0.1**
**TUBD1**	1.546	1.331	1.159	1.666	1.582	1.564	**0.1**
**TUBE1**	2.796	2.553	2.649	3.619	3.511	3.745	**0.1**
**TUBG1**	12.594	13.157	13.373	9.236	9.362	9.060	**0.1**
**TUBG2**	4.222	4.837	4.611	4.539	5.471	5.364	**0.4**

<u>The following references correspond to the legend of Appendix Figure S2:</u>

Reference 1 – Hypoxia regulates HIF-1A
(Semenza, 1999)
Reference 2 – Hypoxia regulates EGR1
(Zhang et al, 2007)
Reference 3 – Hypoxia regulates RBPJ
(Onishi et al, 2016)
Reference 4 – EGR1 regulates HIF-1A
(Sperandio et al, 2009)
Reference 5 – EGR1 regulates NDR1
(Zhang et al, 2007)
Reference 6– ADM - adrenomedullin
(Cormier-Regard et al, 1998; Uemura et al, 2011)
Reference 7– PH4B
(Bentovim et al, 2012)
Reference 8 – LOX-L2
(Schietke et al, 2010; Wong et al, 2011)
Reference 9– PLOD2
(Eisinger-Mathason et al, 2013; Gilkes et al, 2013)
Reference 10– PDK1
(Bentovim et al, 2012; Semba et al, 2016)
Reference 11– TGFBR2
(Wierenga et al, 2014)
Reference 12– STC1
(Ma et al, 2015)
Reference 13– HIf -1A and Notch1
(Pistollato et al, 2010)
Reference 14 - Notch1 may enhance HIF-1A by sequestering HIF-1A away from HIF-1A
(Mahon et al, 2001)
http://www.uniprot.org/uniprot/P46531
Reference 15 – RBJP (also known as CBF1) is downstream Notch1
(Hsieh et al, 1996)
Reference 16 - RBJP inhibits HIF-1A
(Diaz-Trelles et al, 2016)

- Bentovim L, Amarilio R, Zelzer E (2012) HIF1alpha is a central regulator of collagen hydroxylation and secretion under hypoxia during bone development. *Development* **139:** 4473-4483
- Cormier-Regard S, Nguyen SV, Claycomb WC (1998) Adrenomedullin gene expression is developmentally regulated and induced by hypoxia in rat ventricular cardiac myocytes. *The Journal of biological chemistry* **273:** 17787-17792
- Diaz-Trelles R, Scimia MC, Bushway P, Tran D, Monosov A, Monosov E, Peterson K, Rentschler S, Cabrales P, Ruiz-Lozano P, Mercola M (2016) Notch-independent RBPJ controls angiogenesis in the adult heart. *Nature communications* **7:** 12088
- Eisinger-Mathason TS, Zhang M, Qiu Q, Skuli N, Nakazawa MS, Karakasheva T, Mucaj V, Shay JE, Stangenberg L, Sadri N, Pure E, Yoon SS, Kirsch DG, Simon MC (2013) Hypoxia-dependent modification of collagen networks promotes sarcoma metastasis. *Cancer discovery* **3:** 1190-1205
- Gilkes DM, Bajpai S, Chaturvedi P, Wirtz D, Semenza GL (2013) Hypoxia-inducible factor 1 (HIF-1) promotes extracellular matrix remodeling under hypoxic conditions by inducing P4HA1, P4HA2, and PLOD2 expression in fibroblasts. *The Journal of biological chemistry* **288:** 10819-10829
- Hsieh JJ, Henkel T, Salmon P, Robey E, Peterson MG, Hayward SD (1996) Truncated mammalian Notch1 activates CBF1/RBPJk-repressed genes by a mechanism resembling that of Epstein-Barr virus EBNA2. *Molecular and cellular biology* **16:** 952-959
- Ma X, Gu L, Li H, Gao Y, Li X, Shen D, Gong H, Li S, Niu S, Zhang Y, Fan Y, Huang Q, Lyu X, Zhang X (2015) Hypoxia-induced overexpression of stanniocalcin-1 is associated with the metastasis of early stage clear cell renal cell carcinoma. *Journal of translational medicine* **13:** 56
- Mahon PC, Hirota K, Semenza GL (2001) FIH-1: a novel protein that interacts with HIF-1alpha and VHL to mediate repression of HIF-1 transcriptional activity. *Genes & development* **15:** 2675-2686
- Onishi H, Yamasaki A, Kawamoto M, Imaizumi A, Katano M (2016) Hypoxia but not normoxia promotes Smoothened transcription through upregulation of RBPJ and Mastermind-like 3 in pancreatic cancer. *Cancer letters* **371:** 143-150
- Pistollato F, Rampazzo E, Persano L, Abbadi S, Frasson C, Denaro L, D'Avella D, Panchision DM, Della Puppa A, Scienza R, Basso G (2010) Interaction of hypoxia-inducible factor-1alpha and Notch signaling regulates medulloblastoma precursor proliferation and fate. *Stem cells* **28:** 1918-1929
- Schietke R, Warnecke C, Wacker I, Schodel J, Mole DR, Campean V, Amann K, Goppelt-Struebe M, Behrens J, Eckardt KU, Wiesener MS (2010) The lysyl oxidases LOX and LOXL2 are necessary and sufficient to repress E-cadherin in hypoxia: insights into cellular transformation processes mediated by HIF-1. *The Journal of biological chemistry* **285:** 6658-6669
- Semba H, Takeda N, Isagawa T, Sugiura Y, Honda K, Wake M, Miyazawa H, Yamaguchi Y, Miura M, Jenkins DM, Choi H, Kim JW, Asagiri M, Cowburn AS, Abe H, Soma K, Koyama K, Katoh M, Sayama K, Goda N, Johnson RS, Manabe I, Nagai R, Komuro I (2016) HIF-1alpha-PDK1 axis-induced active glycolysis plays an essential role in macrophage migratory capacity. *Nature communications* **7:** 11635
- Semenza GL (1999) Regulation of mammalian O2 homeostasis by hypoxia-inducible factor 1. *Annual review of cell and developmental biology* **15:** 551-578
- Sperandio S, Fortin J, Sasik R, Robitaille L, Corbeil J, de Belle I (2009) The transcription factor Egr1 regulates the HIF-1alpha gene during hypoxia. *Molecular carcinogenesis* **48:** 38-44
- Uemura M, Yamamoto H, Takemasa I, Mimori K, Mizushima T, Ikeda M, Sekimoto M, Doki Y, Mori M (2011) Hypoxia-inducible adrenomedullin in colorectal cancer. *Anticancer Res* **31:** 507-514
- Wierenga AT, Vellenga E, Schuringa JJ (2014) Convergence of hypoxia and TGFbeta pathways on cell cycle regulation in human hematopoietic stem/progenitor cells. *PloS one* **9:** e93494
- Wong CC, Gilkes DM, Zhang H, Chen J, Wei H, Chaturvedi P, Fraley SI, Wong CM, Khoo US, Ng IO, Wirtz D, Semenza GL (2011) Hypoxia-inducible factor 1 is a master regulator of breast cancer metastatic niche formation. *Proceedings of the National Academy of Sciences of the United States of America* **108:** 16369-16374
- Zhang P, Tchou-Wong KM, Costa M (2007) Egr-1 mediates hypoxia-inducible transcription of the NDRG1 gene through an overlapping Egr-1/Sp1 binding site in the promoter. *Cancer research* **67:** 9125-9133
