# Supplementary material for: Tamoxifen mechanically reprograms the tumor microenvironment via HIF‐1A and reduces cancer cell survival
Source: EMBO Rep. 2018 Dec 12;20(1):e46557. doi: 10.15252/embr.201846557 (PMC6322388; doi:10.15252/embr.201846557)
Supplement: Supplementary file 2 — Expanded View Figures PDF [file EMBR-20-e46557-s002.pdf]

Expanded View Figures

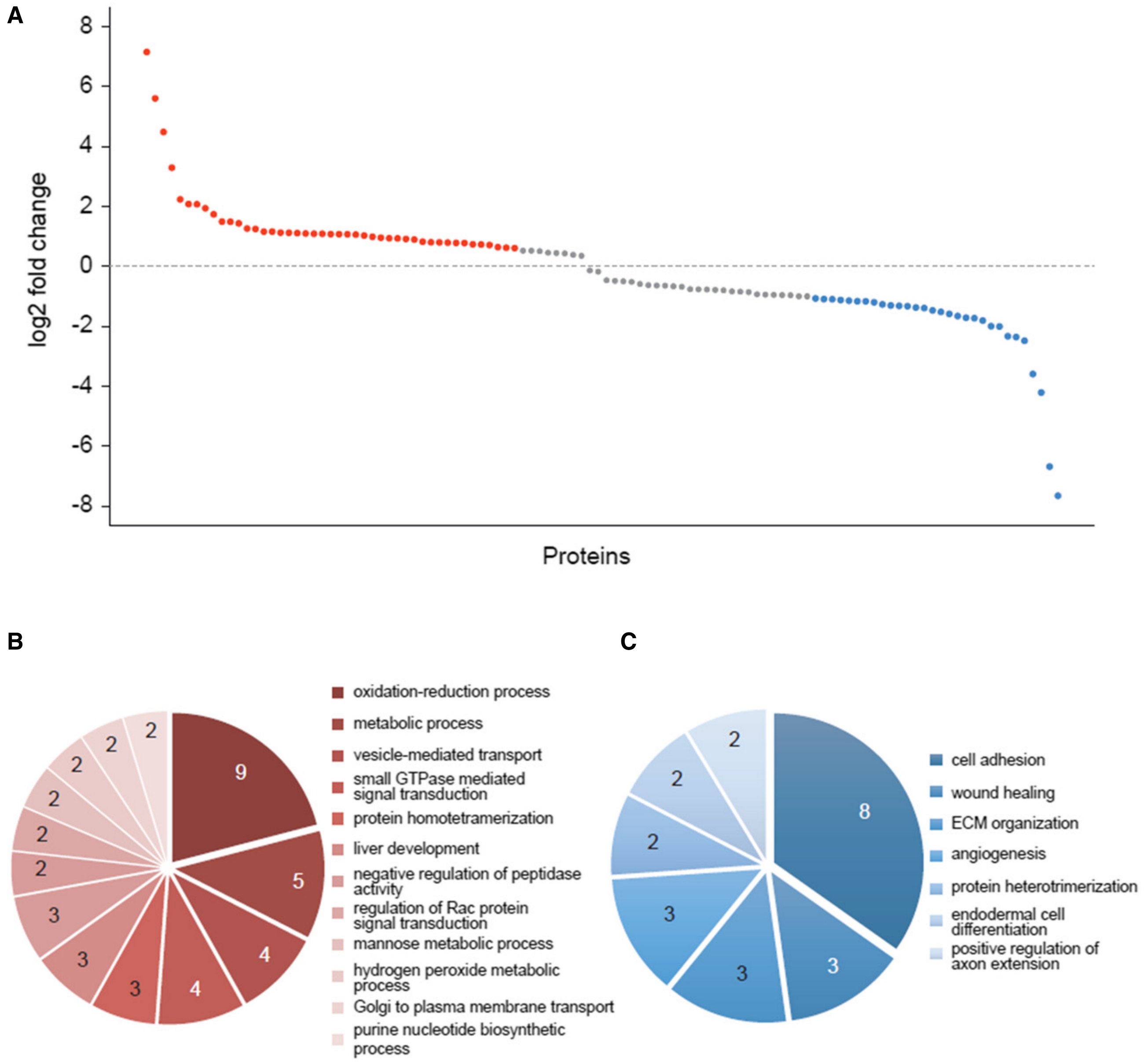

Figure EV1. Tissue proteomics—the tumors in KPC mice treated with 2 mg tamoxifen show changes in their protein content.

A In total, 110 proteins show statistically significant ($P < 0.05$) changes, of which 45 are upregulated (by 50%; red) and 30 downregulated (by 50%; blue).

B, C Enriched ($P < 0.05$) Gene Ontology Biological Processes (GO-BP) for proteins upregulated (B) and downregulated (C) by the tamoxifen treatment.

© 2018 The Authors

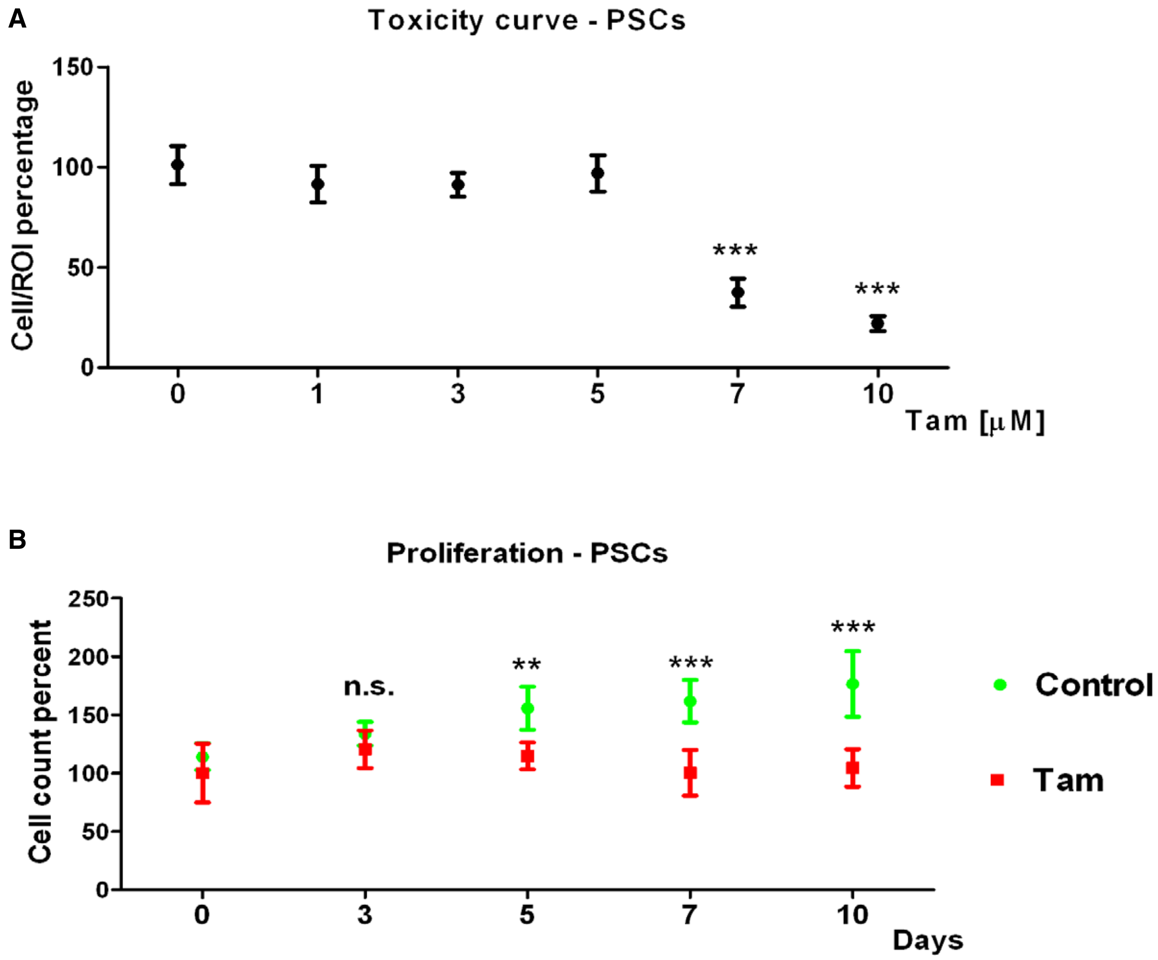

Figure EV2. Toxicity curve and proliferation of PSCs under tamoxifen treatment.

A Killing curve for tamoxifen doses.

B Quantification of cell counting percent relative to time 0—PSCs proliferation.

Data information: Error bars are SEM. $^{**}P < 0.01$, $^{***}P < 0.0001$, n.s. is not significant, *t*-test. $N = 3$ experimental replicates and more than 15 fields of view analyzed per condition.

 © 2018 The Authors

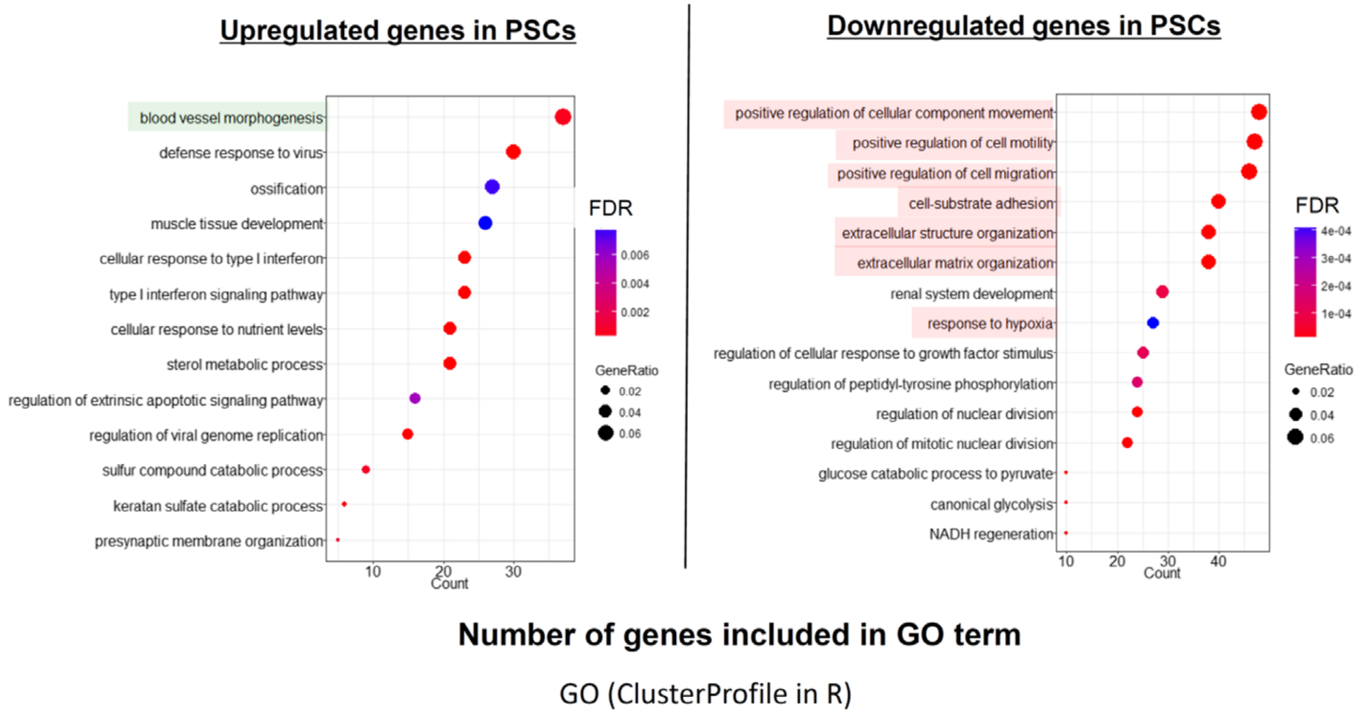

Figure EV3. RNA Sequencing of PSCs—Gene ontology analysis of differentially expressed genes—DEG (upregulated and downregulated).

13 downregulated and 15 upregulated GO terms selected based on the number of DEGs included in each GO term are shown. Color and circle size represent false discovery rate (FDR) and fraction of DEGs assigned to each GO term to total number of DEGs, respectively.
